# Effect of COVID-19 pandemic on the incidence of acute diarrheal disease and pneumonia among under 5 children in Ethiopia- A database study

**DOI:** 10.1371/journal.pgph.0000304

**Published:** 2023-06-14

**Authors:** Abebe Negsso, Balew Arega, Fekadu Abdissa, Brook Zewdu, Ayele Teshome, Abrham Minda, Asnake Agunie

**Affiliations:** 1 Ministry of Health, Addis Ababa, Ethiopia; 2 Yekatit 12 Hospital Medical College, Addis Ababa, Ethiopia; University of Ilorin, NIGERIA

## Abstract

COVID-19 has had a devastating impact on preventable and treatable pediatric diseases in Ethiopia. This study looks at the impact of COVID-19 on pneumonia and acute diarrheal diseases in the country, as well as the differences between administrative regions. In Ethiopia, we conducted a retrospective pre-post study to assess the impact of COVID-19 on children under the age of five who had acute diarrhea and pneumonia and were treated in health facilities during the pre-COVID-19 era (March 2019 to February 2020) and the COVID-19 era (March 2020 to February 2021). From the National Health Management District Health Information System (DHIS2, HMIS), we retrieved data on total acute diarrheal disease and pneumonia, along with their regional and monthly distribution. We calculated incidence rate ratios comparing the rates of acute diarrhea and pneumonia during the pre-and post-COVID-19 eras and adjusted for the year, using Poisson regression. The number of under-five children treated for acute pneumonia decreased from 2,448,882 before COVID-19 to 2,089,542 ((14.7% reduction (95%CI;8.72–21.28), p<0.001)) during COVID-19. Similarly, the number of under-five children treated for acute diarrheal disease decreased from 3,287,850 in pre-COVID-19 to, 2,961,771((9.91% reduction (95%CI;6.3–17.6%),p<0.001)) during COVID-19. In the majority of the administrative regions studied, pneumonia and acute diarrhea diseases decreased during COVID-19, but they increased in Gambella, Somalia, and Afar. During the COVID-19 period, the greatest reduction of children with pneumonia (54%) and diarrhea disease (37.3%) was found in Addis Ababa (p<0.001). The majority of administrative regions included in this study have seen a decrease in pneumonia and acute diarrheal diseases among children under the age of five, while three regions namely, Somalia, Gambela, and Afar saw an increase in cases during the pandemic. This emphasizes the importance of using tailored approaches in mitigating the impact of infectious diseases such as diarrhea and pneumonia during situations of a pandemic such as COVID-19.

## 1. Introduction

Acute diarrhea and pneumonia are leading causes of morbidity and mortality in children under the age of five worldwide, especially in Sub-Saharan Africa and South Asia [1]. Globally, 1.23 million children **die** of pneumonia and diarrhea before reaching their 5^th^ birthday—the equivalent of over 141 child deaths per hour or 3,400 deaths per day [2]. They share several risk factors and can be avoided by implementing proven interventions such as improved personal hygiene and routine vaccinations. Prompt detection and treatment can aid in the avoidance of complications. The current COVID-19 pandemic has not only posed a challenge, but also an opportunity to observe the several pandemic control measures implemented to bring down the transmission of these diseases and, as a result, under-five mortality [3].

With reference to the Millennium Development Goal 4 (MDG 4), child survival has improved significantly globally. It was planned to reduce the under-five mortality rate by two-thirds, between 1990 and 2015 [4]. Currently, the Sustainable Development Goals (SDGs 3.2) are being implemented in various nations including Ethiopia to reduce child mortality to less than 25 per 1000 by 2035 [5]. COVID-19 has threatened to undo the progress made thus far, casting doubt on the SDG targets due to its effect on socioeconomic issues, nutrition, and disruption of basic healthcare delivery systems [6]. Because 88% of diarrhea and pneumonia cases among under-five children are due to inadequate hygiene, COVID-19 prevention precautions such as mandatory mask-wearing and hand hygiene practices have undoubtedly added to a reduction in the incidence of these preventable diseases in the community [7]. However, two out of every five people lack access to basic hand-washing facilities, mostly in developing countries [8]. This makes it difficult for people to fully adhere to COVID-19 preventative measures like hand washing.

Ethiopia is one of the top five nations in the world for childhood pneumonia and diarrheal disease, with only three out of ten children under the age of five receiving treatment [9]. Keeping in line with the Sustainable Development Goals (SDG), under-five mortality must be reduced by more than half to meet the SDG target [10]. Even though the country has been able to reduce this problem by two-thirds in the past decade, under-five child mortality, which is primarily caused by diarrhea and pneumonia, remains a serious issue in the country [11]. In addition, the ongoing COVID-19 pandemic is expected to worsen the problem. So, this is crucial to the assessment of the actual nature of the damage.

Ethiopia has the most confirmed COVID-19 cases in East Africa (149,689) as of February 2021, with reported COVID-19 cases varying by administrative region [12]. As of February 2022, Addis Ababa had the most COVID-19 cases. In resource-limited countries like Ethiopia, children disproportionately suffered from the pandemic’s indirect effects. In a prospective vaccination card review study in northern Ethiopia, age-eligible vaccination coverage among children during the COVID-19 outbreak was 12.5% lower compared to the pre-COVID time [13]. However, no extensive data on the impact of COVID-19 on prevalent childhood diseases such as diarrhea and pneumonia in Ethiopia is available. Here, we have used secondary data (health management information system) collected from health institutions in the country to assess the impact of the COVID-19 pandemic on under-five children’s diarrheal disease and pneumonia across different regions. We further investigated monthly trends of diarrhea and pneumonia in the epicenter of COVID-19, Addis Ababa.

## 2. Methods

### 2.1 Study design

We conducted a retrospective pre-and post-COVID-19 study using health management information system data to assess the impact of COVID-19 on diarrheal and pneumonia diseases treated at health facilities before (March 2019-February 2020) and during the pandemic (March 2020-February 2021).

### 2.2 Study setting

Ethiopia is the second-most populous country in Africa, with an estimated population size of over 115 million in 2021 [14]. The country has ten administrative regions, which are: Tigray, Afar, Amhara, Oromia, Somalia, Sidama, Benishangul-Gumuz, Harari, Gambella, and the Southern Nations, Nationalities, and Peoples’ Region (SNNPR), and two city administrations (Addis Ababa and Dire-Dawa). According to the most recent national EDHS (2016), more than a third of the population in Afar, Somalia, SNNPR, and Amhara travel more than 30 minutes round-trip for household water. Only 13% of homes have soap and water for handwashing, 93% of households cook with solid fuel, and only 47% of households have separate cooking buildings [15]. The Ethiopian government had put in place some steps to stop COVID-19 from spreading across the country, including handwashing with soap and water or alcohol-based hand rub and universal mask use (Table 1).

**Table 1:** Hand hygiene and mask use guidelines to prevent COVID-19 in Ethiopia.

Cough hygiene	Universal mask-wearing should be practiced by everyone. The type of mask use is based on the level of risk of acquiring COVID-19.
Material &procedure	• Proper mask utilization is any medical, non-medical, respirator, or cloth mask, which covers the nose, mouth, and chin of an individual. • The public should implement cough hygiene, including covering the mouth during coughing and sneezing with a tissue or flexed elbow.
Hand hygiene	
Access	• It should provide universal access to hand hygiene facilities in front of public buildings and transport hubs- such as markets, shops, places of worship, schools, and train or bus stations
Material and procedure	• Hands with visible dirt should be washed with soap and water for 40–60 seconds. • Hands without visible dirt can be cleaned with an alcohol-based hand rub (based on 80% alcohol) for 20–30 seconds.

The Addis Ababa city, Oromia, and Amhara regions reported the majority of COVID-19 cases, while Gambella, Somalia, and Afar reported the least (Table 2). All regions are included in this analysis, except Tigray, which has been under conflict since November 2020, and Sidamo, which is a newly constituted region

**Table 2:** The distribution of COVID-19 cases across the administrative regions in Ethiopia as of February 28/ 2021.

Area	Total number of COVID-19 cases (%)
Addis Ababa city	98,046 (65.5)
Oromia region	20,956 (14.0)
Amhara region	6,137 (4.1)
SNNPR region	4,790 (3.2)
Dire Dawa city	2,994 (2.0)
Hareri region	2,395 (1.6)
Benshangul Gumz	1,946 (1.3)
Afar region	1,497 (1.0)
Somalia region	1,347 (0.9)
Gambela	748 (0.5)
**Total number of COVID-19 cases**	149,689 (94.1)

### 2.3 Study population, inclusion, and exclusion criteria

All under-five children who were treated for acute diarrheal diseases and pneumonia in the health facilities during the study periods were the study population. Children with missing information including child age, months, and/or year of treatment were excluded from the study.

### 2.4 Data source and variables

We used data generated from the National Health Management District Health Information System (DHIS2 HMIS) between March 2019 and February 2021. We included data on the Hospital/Clinic Monthly Service Delivery Report based on the priorities of the Plan for Accelerated and Sustained Development to end poverty. We divided the data into two periods: the COVID-19 era (March 2020 to February 2021 and above) and the Pre-COVID-19 era (March 2019 to February 2020). We collected data based on the national DHIS2 HIMs indicators for diarrheal disease and pneumonia reported every month. Specifically, we extracted data that included a year of study(months), years, region, total pneumonia cases, and total diarrheal cases treated using oral rehydration solution with or without Zinc. The proportion of under-five children treated for diarrhea and pneumonia during the COVID-19 and pre-COVID-19 periods was the outcome variable studied.

### 2.5 Operational definitions

**Diarrhea**: defined as three or more loose or liquid stools per day (or more frequent passage than is normal for the individual). When diarrhea lasts less than 14 days, it is referred to as acute, and when it lasts more than 14 days, it is referred to as chronic or persistent [16].

**Pneumonia**: is defined as an infection of one or both of the lungs caused by bacteria, viruses, or fungi [17].

### 2.6 Data analysis, sources of data, and data collection

We used Stata (ver. 16) software to analyze the data [18]. We used descriptive analysis to describe the counts across the regions. Between the COVID-19 and pre-COVID-19 eras, we compared the rate of pneumonia and diarrhea cases treated in health facilities, the difference across the administrative regions, and study months. We used poison regression to estimate the difference between the COVID-19 era and the pre-COVID-19 period per month. In Poisson regression, we adjusted for the month. A 95% confidence interval (CI) with a level of significance set at 5% (P <0.05) was considered.

### 2.7 Ethical considerations

Since this was a secondary data analysis based on an existing database, a waiver of written informed consent from participants was obtained.

## 3. Result

### 3.1 Overall number of pneumonia and acute diarrhea diseases

We looked at DHIS2 HMIS data over 24 months (pre-COVID-19: March 2019-February 2020 and COVID-19: March 2020-February 2021) for this study. There were 2,448,882 cases of under-five pneumonia in the 12 months pre-COVID-19. During the COVID-19 pandemic, there were 2,089,542 cases of under-five children pneumonia reported to health centers during the next 12 months. This is a reduction of 359,340 instances (14.7%, CI (8.72–21.28) reduction, p<0.001). Similarly, there were 3,287,850 cases of under-five children diarrheal disease in the 12 months of pre-COVID-19. During the COVID-19 pandemic, there were 2,961,771 cases of under-five children diarrheal disease reported to health centers during the next 12 months. This resulted in a reduction of 326,079 instances (9.9%, CI (6.3–17.6) reduction, p<0.001). Table 3 shows the total number of instances recorded across the administrative region.

**Table 3:** Shows the number and distribution of pneumonia and acute diarrheal disease among under-five children in Ethiopia’s administrative regions from March 2019 to February 2021.

Administrative regions	Pneumonia			acute diarrhea disease		
	Pre-COVID-19	COVID-19	Change during COVID-19	Pre-COVID-19	COVID-19	Change during COVID-19 N(%)
	N	N	N(%)	N	N	
Oromina	1336123	1068848	-267275(20.0)	1521269	1409682	-111587(7.34)
Amhara	445972	410534	-35438 (7.95)	678306	639046	-39260(5.79)
Addis Ababa	54665	23809	-30856 (56.45)	102118	73005	-29113 (28.51)
SNNPR	406997	373888	-33109 (8.13)	780476	526541	-253935(32.54)
Benshangul Gumze	36193	29867	-6326(-17.48)	57214	56423	-791(1.38)
Somallia	121482	126001	+4519(3.59)	137911	179614	+41703 (23.22)
Dire Dawa	4787	3740	-1047(21.87)	11652	8920	-2732 (23.45)
Hareri	4922	6462	-1540(31.29)	7498	6689	-809 (10.79)
Gambella	9147	9597	+450(4.69)	10346	14231	+3885 (27.30)
Afar	28594	36797	+8203(22.29)	33402	47621	+14219(29.86)
Total cases	2448882	2089542	-359340(14.67)	3287850	2961771	-326079(9.92)

^**-**^ Decrease of number during COVID-19, ^+^ Increase of number during COVID-19

### 3.2 National level: Pattern of pneumonia and diarrheal diseases

**Fig 1:**
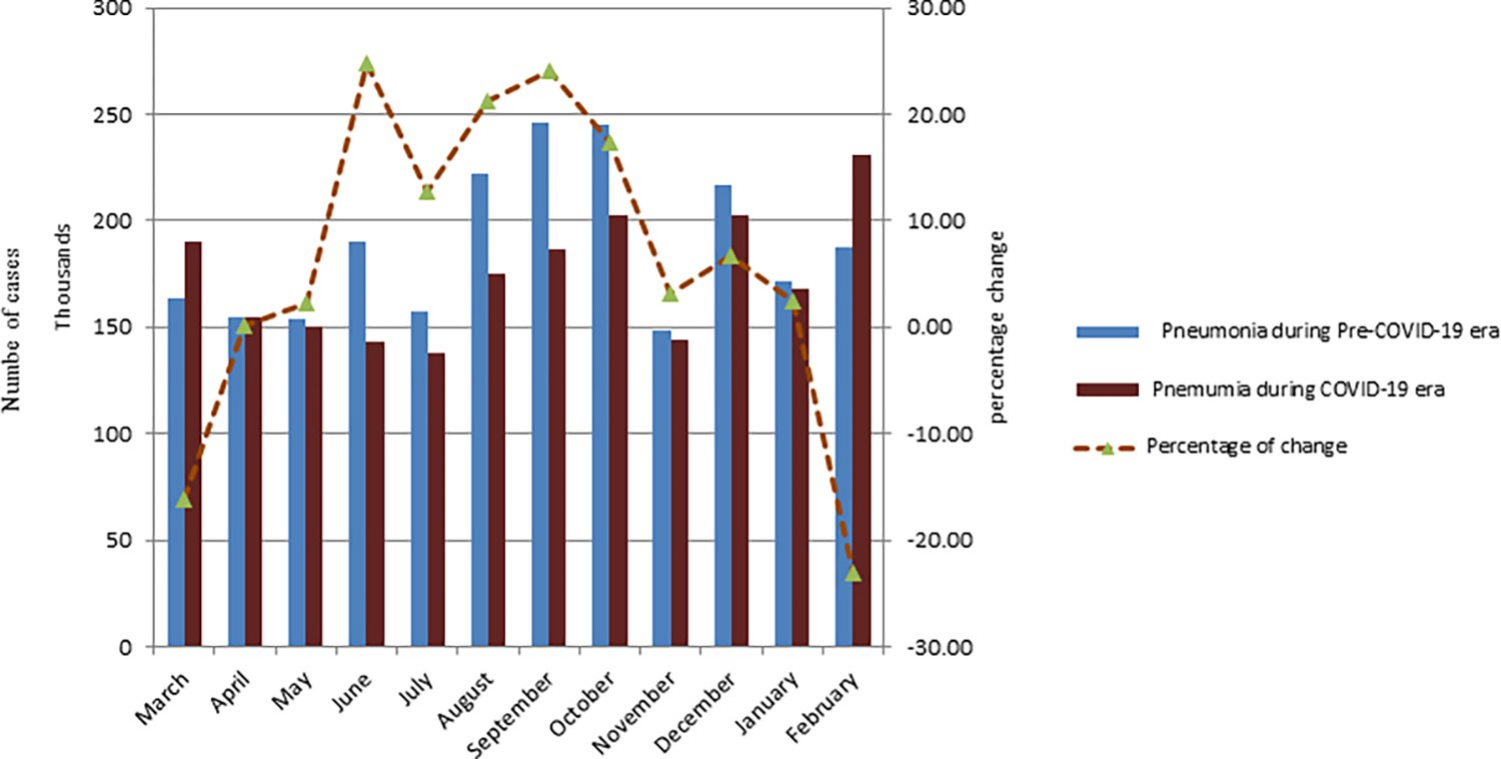
Average number of under-five children treated for pneumonia at health facilities per month during the study periods in Ethiopia.

During the COVID-19 era, there was a 15% reduction in under-five children treated for pneumonia at health facilities (rate ratio = 0.85, p<0.001) compared to pre-COVID-19(244,882 versus 2089542). From June to October, we found the highest decline in pneumonia cases (Fig 1).

**Fig 2:**
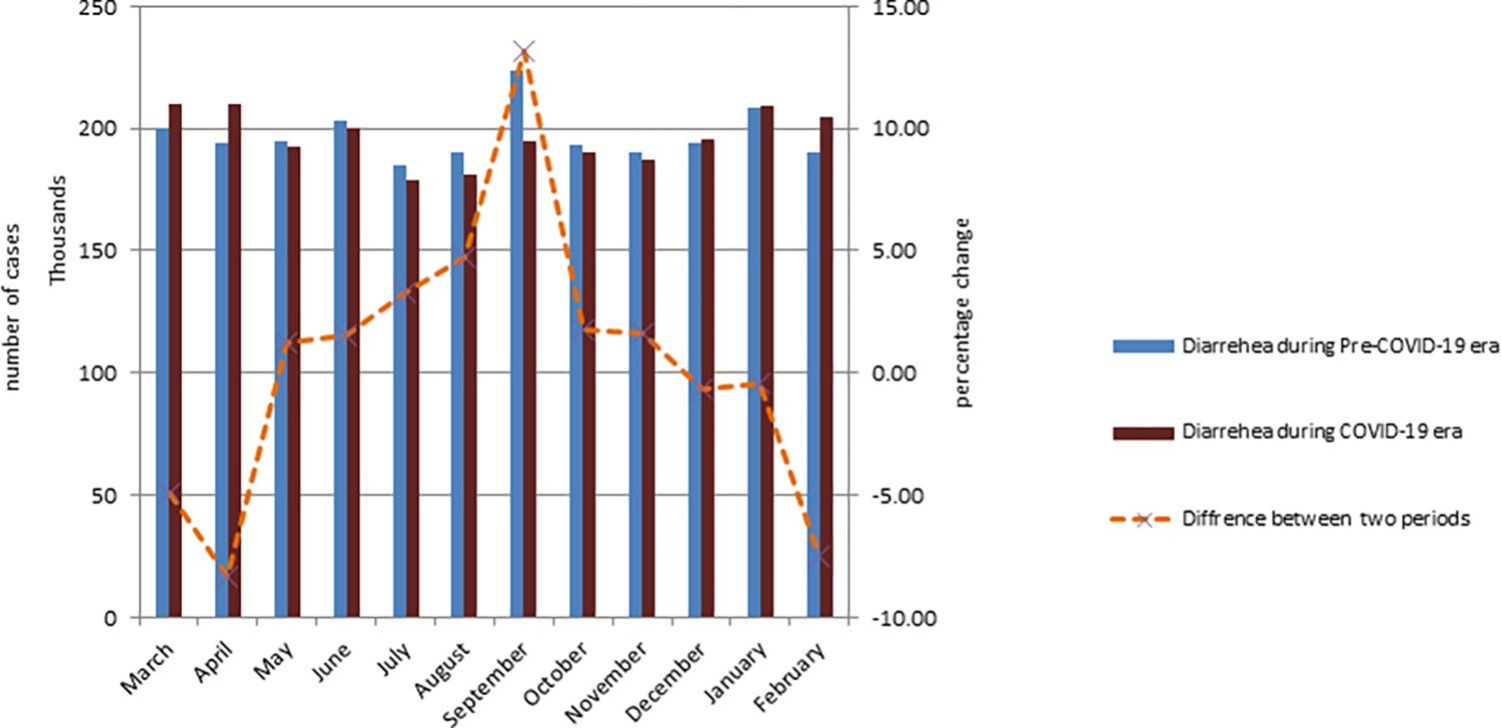
Average number of under-five children treated for acute diarrhea disease at health facilities per month during the study periods in Ethiopia.

In under-five children with acute diarrhea treated at health facilities throughout the COVID-19 era, there was a 10% reduction (328,850 versus 296,1771) in comparison to pre-COVID-19 (rate ratio = 0.90, p<0.001). From July to September 2020, we found the lowest rate of pneumonia (Fig 2).

### 3.3 Regional level: Pattern pneumonia and diarrheal diseases

**Fig 3:**
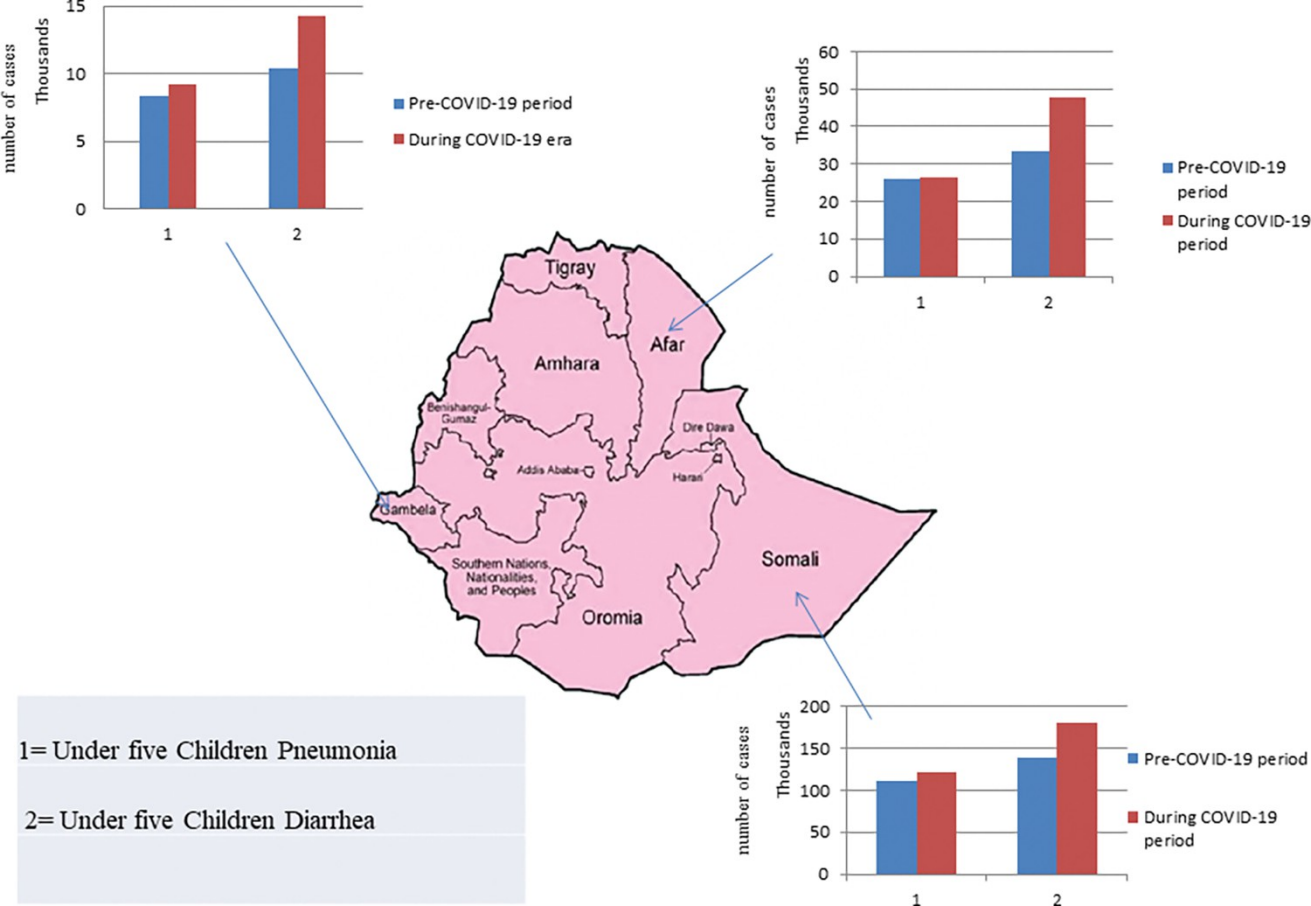
Rate of reduction of pneumonia and acute diarrheal disease across different administrative regions among under-five children during the study periods (Base layer map is adapted from a previous study (19)).

During the COVID-19 period, the trend of pneumonia and diarrhea disease among under-five children decreased in most regions. During the COVID-19 era, pneumonia declined in seven of the 12 regions studied, ranging from 3% in SNNPR to 54.2% in Addis Ababa city. Acute diarrhea disease also decreased, with rates from 3.5%in Amhara to 45% in Harer during the COVID-19 era (Fig 3).

**Fig 4:**
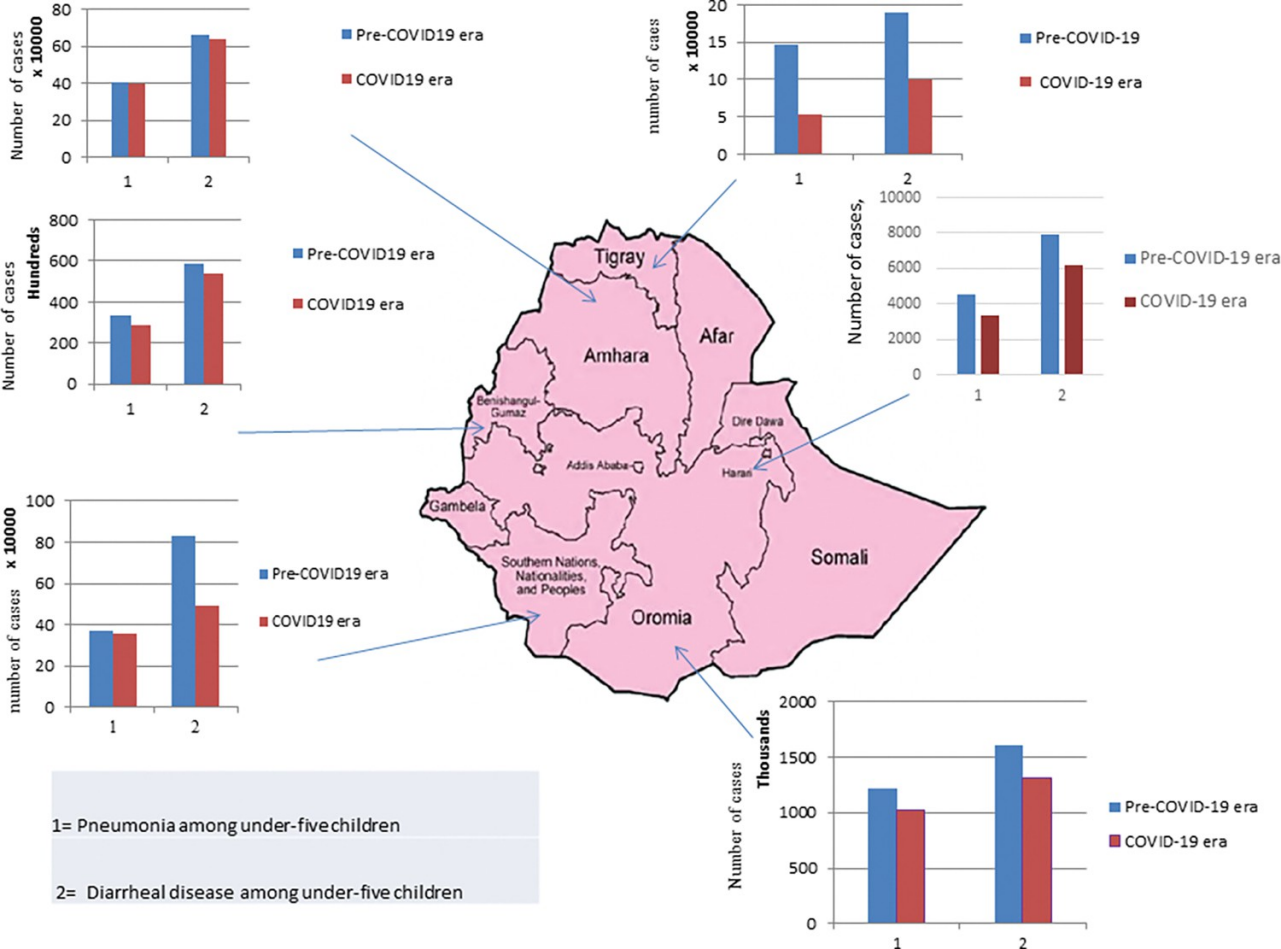
Rate of increase of pneumonia and acute diarrheal disease across different administrative regions among under-five children during the study periods (Base layer map is adapted from a previous study (19)).

Pneumonia and acute diarrhea diseases increased during the COVID-19 era compared to the pre-COVID-19 period in the remaining three regions, namely Somalia, Gambela, and Afar. Pneumonia cases increased from 1% in Afar to 10% in Gambela, and acute diarrhea disease increased from 23.3% in Somalia to 29% in Afar (Fig 4).

### 3.4 Addis Ababa level: Pattern pneumonia and diarrheal diseases

**Fig 5:**
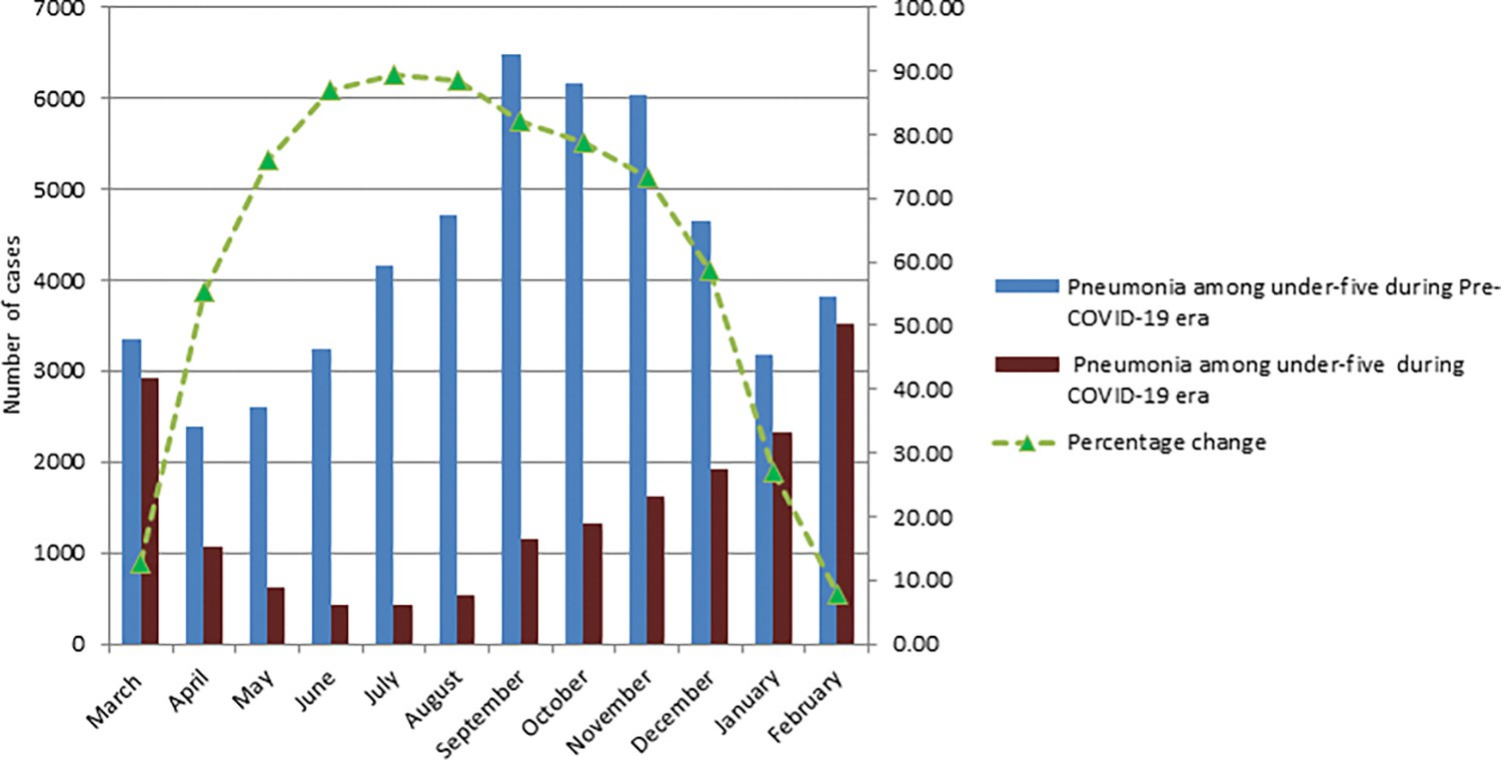
Average number of under-five children treated for pneumonia at health facilities in Addis Ababa per month during the study periods in Addis Ababa.

During the COVID-19 era, we found that the number of under-five children treated at health institutions for pneumonia reduced by 54% (50773 vs 17885) (rate ratio = 0.46 p0.001). When compared to the pre-COVID-19 months, the average monthly reported cases of pneumonia was reduced by more than half from April to December 2020 (Fig 5).

**Fig 6:**
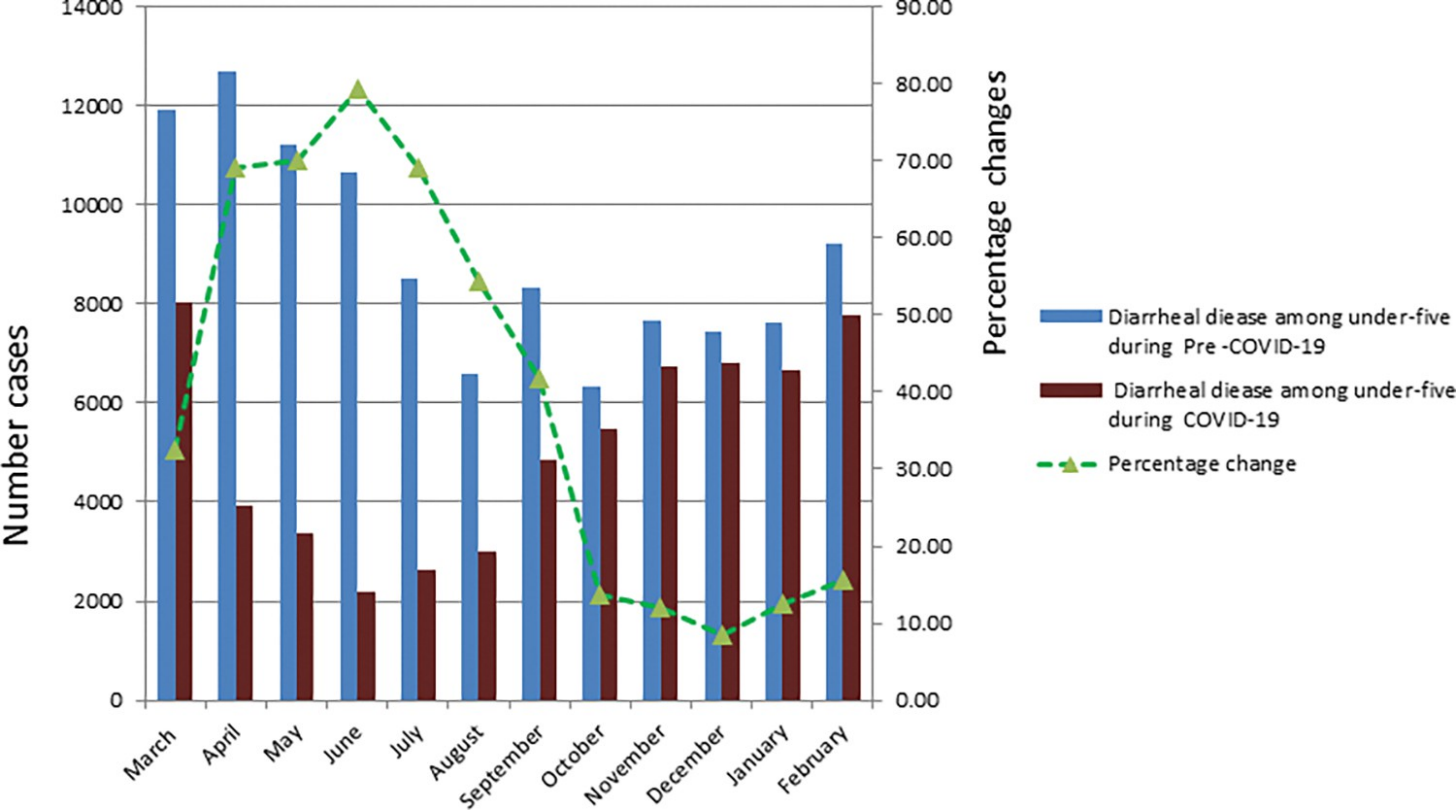
Average number of under-five children treated for acute diarrhea disease at health facilities per month during the study period in Addis Ababa.

During the same study period, diarrheal diseases treated during COVID-19 periods decreased by 37.3% (108240 versus 61456) (rate ratio = 0.627 p<0.001). Between April and August 2020, the average monthly reported cases of diarrheal diseases decreased by more than 50% compared to the pre-COVID-19 months (Fig 6).

## 4. Discussion

This study summarizes the evidence on the impact of COVID-19 on pneumonia and acute diarrheal diseases under-five children who were treated in Ethiopian healthcare facilities during COVID-19. The findings reveal a reduction in the number of diseases in under-five children treated at health facilities for pneumonia and acute diarrheal diseases over the COVID-19 period, with differences across the country’s administrative regions.

This study found that during the COVID-19 period (March 2020–February 2021), there was a significant reduction in cases of pneumonia and acute diarrheal diseases treated at health facilities at the national level. Considering the bright side, a decrease in the incidence of these preventable diseases had a significant impact on reducing the mortality rate among under-five children. On the other hand, COVID-19’s indirect effects, which are linked to poor access to health services, are expected to account for 40% of all additional child deaths globally [19]. In Ethiopia, particularly during the first six months of the pandemic, there was widespread public fear of contracting the disease from healthcare facilities, resulting in a reduction in the number of people seeking medical attention. Parents may be forced to care for their sick children at home as a result of these circumstances. For example, pneumonia significantly dropped more than diarrhea, indicating that the family is worried about COVID-19-related cough and may keep the baby at home. A previous study in Ethiopia found a 35% decline in under-five children’s services [20], and a significant drop in the use of reproductive, maternal, and newborn healthcare services [21]. COVID-19 has been shown to have an effect in previous studies, with 30% and 36% reductions in Sierra Leone and South Africa, respectively [22, 23]. The COVID-19 pandemic could compromise vaccine delivery among under-five children, with an estimated 80 million children globally affected by disrupted immunization services and campaigns [24]. Ethiopia, which already has a low vaccine coverage rate [25], could have been affected by the pandemic. For example, the coverage of the first dose of pentavalent vaccines was lowered in two recent studies undertaken in the country during the pandemic period, one evaluating age-eligible vaccination coverage [26] and the other evaluating the first dose of pentavalent vaccine coverage [27]. As a result, vaccine-preventable illnesses in children, such as pneumonia and diarrhea, intensify during the COVID-19 period. During the pandemic, community members are encouraged to wash their hands with soap and wear masks, which helps to reduce the spread of diarrheal diseases [28] and respiratory infections, respectively [29, 30]. However, in Ethiopia, 43% of the rural population lacks access to improved sources of clean water, making regular hand-washing practices difficult during COVID-19 [31]. Furthermore, investigations conducted during COVID-19 revealed decreased adherence to the COVID-19 preventive actions that were advised [32–34]. Given the aforementioned factors and challenges, the decrease in pneumonia and diarrhea we observed could be due to the pandemic’s indirect detrimental impact. This has a long-term negative impact on children’s well-being, as well as reversing hard-won progress in achieving SDG 3.2 targets [35]. This highlights the importance of reducing COVID-19’s detrimental impact on children and achieving universal health coverage targets for child health services post-pandemic period.

We found considerable regional disparities in pneumonia and diarrhea disease among under-five children treated at health facilities across administrative regions during COVID-19. We found an increased report of these diseases in the Gambella, Somalia, and Afar regions. These regions are predominantly pastoralist (85%), with most people living in hard-to-reach/remote areas facing developmental inequities. According to the most recent Ethiopia Demographic and Health Survey (2016), more than two-thirds of people in Afar and Somalia found it difficult to find water sources for their households, and less than 1.4% of the population in these regions possessed soap and water for hand hygiene [36]. Previous research found that areas with limited access to water and sanitation facilities have been disproportionately affected by diarrhea and pneumonia diseases; conditions that could have been avoided if effective hygiene had been implemented [37]. In the previous two years, the Ministry of Health, in partnership with regional health, has opened over ten new health posts and trained a large number of health professionals and health extension workers [38]. The first incidence of COVID-19 was found in each of the three regions within a month following the national report, and they were listed as the country’s least three COVID-19 reporting regions [39]. As a result, preventive measures such as hand washing and universal making may not be practiced regularly in these areas. Therefore, the pattern of childhood preventive diseases, such as acute diarrheal disease and pneumonia, was unaffected and continued to rise, as seen in the pre-COVID-19 years.

Since the first few COVID-19 cases were reported in Ethiopia, Addis Ababa has accounted for the vast majority of COVID-19 cases (up to 90%) [40]. As a result, the city is expected to see the greatest impact of the pandemic on crucial health care. During the COVID-19 period, we found a significant reduction in the number of under-five children treated for diarrheal disease and pneumonia in the city. In Addis Ababa, access to safe drinking water and deprivation rates in access to a safe source of drinking water and solid waste disposal are near zero, in stark contrast to the rest of the country [15]. Furthermore, because the city is the epicenter of the pandemic, COVID-19 preventive methods such as handwashing with soap or alcohol-based hand rub and community mask-wearing have been heavily promoted. It appears logical that the most impacted area will be given priority in terms of implementing preventive actions to contain the infection in that area and concomitantly affect the pattern (negatively or positively) of acute diarrheal and pneumonia among under five-year children [41].

This study attempted to understand the impact of COVID-19 on preventable childhood illnesses by using large sample size, multicenter data, and a comparative analysis of routine data to reveal the national picture. However, we were unable to determine individual socio-demographic variables and COVID-19 public health preventative interventions on acute diarrheal disease and pneumonia, as well as trends because we employed aggregated data. In addition, more research is needed to determine if the reduction of these acute illnesses among under-five children is a negative or positive effect of COVID-19 in the areas.

In conclusion, the result of this study revealed that COVID-19 has an effect on common acute illnesses among under-five children. To address these challenges, the SDG program should promptly adjust to the new normal, bolstering the patient-centered approach to child care and community-based child care to reach individuals who have not attended health facilities.

Personal and environmental hygiene practices like hand washing with soap and water, wearing a mask, and alcohol hand rubbing are the main preventive measures against COVID-19 infection. These activities also are important to prevent and control acute diarrheal diseases and pneumonia. To maintain the progress made in achieving the millennium development goal of reducing under-five child mortality over the past ten years and achieving the SDG targets in the country, these measures must be implemented consistently over the post-COVID-19 era.

## Supporting information

S1 File Inclusivity in global research questionnaire. (DOCX)

## References

[1] Aiello AE, Coulborn RM, Perez V et al. Effect of Hand Hygiene on Infectious Disease Risk in the Community Setting: A Meta-Analysis.Am J Public Health 2008; 98:1372–1381. 10.2105/AJPH.2007.124610 PMC2446461 18556606

[2] IVAC at Johns Hopkins Bloomberg School of Public Health. Pneumonia and diarrheal diseases report 2020

[3] Jung S, Doh YA, Bizuneh DB, Beyene H et al. The effects of improved sanitation on diarrheal prevalence, incidence, and duration in children under-five in the SNNPR State, Ethiopia: study protocol for a randomized controlled trial. Trials. 2016;18;17(1):204. 27089872 10.1186/s13063-016-1319-z PMC4835836

[4] Li Liu, Oza SH Hogan DA et al. Global, regional, and national causes of under-5 mortality in 2000–15: an updated systematic analysis with implications for the SDGs. Lancet 2016; 388: 3027–35 27839855 10.1016/S0140-6736(16)31593-8 PMC5161777

[5] United Nations General Assembly. Transforming our world: the 2030 Agenda for Sustainable Development. New York: United Nations, 2015

[6] You D, Hug L, Ejdemyr S, et al. Global, regional, and national levels and trends in under-5 mortality between 1990 and 2015, with scenario-based projections to 2030: a systematic analysis by the UN Inter-agency Group for Child Mortality Estimation. Lancet 2015; 386: 2275–86. doi: 10.1016/S0140-6736(15)00120-8 26361942

[7] WHO. Ending Preventable Child Deaths from Pneumonia and Diarrhea by 2025.

[8] Odo DB, Mekonnen AG Availability and factors influencing community level hand-washing facility in Ethiopia: Implication for prevention of infectious diseases. PLoS ONE 2021; 16(1): e0243228. 33465087 10.1371/journal.pone.0243228 PMC7815131

[9] Ethiopia] and ICF. Ethiopia demographic and health survey 2016. Addis Ababa and Rockville: CSA and ICF; 2016.

[10] Getaye Y, Achaw A. Newborns and Under-5 Mortality in Ethiopia: The Necessity to Revitalize Partnership in Post-COVID-19 Era to Meet the SDG Targets. Journal of Primary Care & Community Health 2021; 12(2): 1–5:1–5 10.1177/2150132721996889 PMC7917850 33632030

[11] FDRE.The 2017 voluntary national reviews on SDGs of Ethiopia: government commitments, national O and performance trends. National Plan Commission; 2017.

[12] Worldometer Whttps://www.worldometers.info/coronavirus/country/Ethiopia. Ethiopia Coronavirus cases: Accessed on March 2021.

[13] Miretu Debrnesh Goshiye, Zinet Abegaz Asfaw & Sisay Gedamu Addis (2021) Impact of COVID-19 pandemic on vaccination coverage among children aged 15 to 23 months at Dessie town, Northeast Ethiopia, 2020, Human Vaccines & Immunotherapeutics, 17:8, 2427–2436. 33721546 10.1080/21645515.2021.1883387 PMC8475620

[14] World bank in Ethiopia:worlhttps://www.worldbank.org/en/country/ethiopia/overview. Accessed in march 2021.

[15] EDHS.Ethiopian Demographic Health Survey, Key Indicators.CSA, ICF; 2016.

[16] Diarrhoeal Disease Fact Sheet N 330. Available at: http://www.who.int/mediacentre/factsheets/fs330/en/. Accessed 16 September 2015.

[17] Mackenzi Grant. The definition and classification of pneumonia. BMC Pneumonia (2016) 8:14 10.1186/s41479-016-0012-z PMC5471962 28702293

[18] Mekonnen A, Assefa T, Tesfaye K and Getahun D. Spatial Distribution of Nitrate in the Drinking Water Sources Found in Ethiopia; Retrospective Study. Glob. J. Environ. Sci. Technol 2014; 2(7): 075–081.

[19] Yihun B, Tadele G, Tebekaw Y, Tesfa B. Immediate Impacts of the COVID-19 Pandemic on Essential Maternal, and Child Health Service Delivery and Practices in Selected Health Facilities in Ethiopia. Conference Paper.August 2020

[20] Kassie A, Wale A, Yismaw W. Impact of Coronavirus Diseases-2019 (COVID-19) on Utilization and Outcome of Reproductive, Maternal, and Newborn Health Services at Governmental Health Facilities in South West Ethiopia, 2020: Comparative Cross-Sectional Study. Int J Women’s Health 2021;13:479–488 doi: 10.2147/IJWH.S309096 34040456 PMC8141395

[21] World Vision Policy Brief: COVID-19 & Risks to Children’s Health and Nutrition. May 2020

[22] Jensen C, McKerrow NH. Child health services during a COVID-19 outbreak in KwaZulu-Natal Province, South Africa. S Afr Med J 2020; 15(0):13185. 33334393

[23] Jones-Konneh TEC, Kaikai AI et al. Impact of health systems reform on COVID-19 control in Sierra Leone: a case study. Trop Med Health. 2023 May 17;51(1):28. doi: 10.1186/s41182-023-00521-z 37198669 PMC10189713

[24] UNICEF. At least 80 million children under one are at risk of diseases such as diphtheria, measles, and polio as COVID-19 disrupts routine vaccination efforts, warn Gavi, and UNICEF, 2020

[25] Nour TY, Farah AM, Ali OM, Abate KH. Immunization coverage in Ethiopia among 12-23-month-old children: systematic review and meta-analysis.BMC Public Health 2020;; 20(1):1134. 10.1186/s12889-020-09118-1 PMC7370412 32689962

[26] Abdela SG, Berhanu AB, Ferede LM, van Griensven J. Essential Healthcare Services in the Face of COVID-19 Prevention: Experiences from a Referral Hospital in Ethiopia. Am J Trop Med Hyg 2020;103(3):1198–1200. doi: 10.4269/ajtmh.20-0464 32762799 PMC7470545

[27] OladejiOl Oladeji BI, FaraAE.Assessment of the Effect of the COVID-19 Pandemic on the Utilization of Maternal Newborn and Child Health Services in the Somali Region of Ethiopia.Journal of Epidemiology and Public Health 2020; 05(04): 458–469

[28] Bloomfield S.F, Aiello AE, and Cookson B. The effectiveness of hand hygiene procedures in reducing the risk of infections in homes and community settings including hand-washing and alcohol-based hand sanitizers. Am. J. Infect. Control 2007; 35, S27–S64.).

[29] MacIntyre CR, Cauchemez S, Dwyer DE, Seale H, Cheung P, Browne G, et al. Face mask use and control of respiratory virus transmission in households. Emerg Infect Dis 2009;15(2):233–41.). doi: 10.3201/eid1502.081167 19193267 PMC2662657

[30] Koul PA, Mir H, Saha S, et al. Respiratory viruses in returning Hajj and Umrah pilgrims with acute respiratory illness in 2014–2015. Indian J Med Res 2018;148:329–33 30425224 10.4103/ijmr.IJMR_890_17 PMC6251276

[31] Bante A, Mersha A, Tesfaye et al. Adherence with COVID-19 Preventive Measures and Associated Factors Among Residents of Dirashe District, Southern Ethiopia. Patient Prefer Adherence 2021;15:237–249 doi: 10.2147/PPA.S293647 33568900 PMC7868191

[32] Shewasinad YS, Asefa KK, Mekonnen AG, et al. Predictors of adherence to COVID-19 prevention measure among communities in North Shoa Zone, Ethiopia based on health belief model: A cross-sectional study. PLoSONE 2021;16(1): e0246006 10.1371/journal.pone.0246006 PMC7822535 33481962

[33] Azene ZN, Merid MW, Muluneh AG. Adherence toward COVID-19 mitigation measures and its associated factors among Gondar City residents: A community-based cross-sectional study in Northwest Ethiopia. PLoS ONE 2020; 15(12): e0244265 33378332 10.1371/journal.pone.0244265 PMC7773181

[34] WHO/UNICEF. Hygiene Baselines pre-COVID-19 Global Snapshot. 2020

[35] World Health Organization.World Health Statistics 2016: Monitoring Health for the SDGs. World Health Organization; 2016

[36] CSA. Ethiopia Demographic and health survey 2016. Addis Ababa, Ethiopia, and Rockville, Maryland, USA, 2016.

[37] McGuinness SL, Barker S.F,Toole J. Effect of hygiene interventions on acute respiratory infections in childcare, school and domestic settings in low- and middle-income countries: a systematic review. Trop Med Int Health 2018; 23(8):816–33. 29799658 10.1111/tmi.13080

[38] Mohammed H, Oljira L, Roba KJ. Containment of COVID-19 in Ethiopia and implications for tuberculosis care and research. Infect Dis Poverty 2020; 9: 131. doi: 10.1186/s40249-020-00753-9 32938497 PMC7492795

[39] Assefa Y., Gelaw Y.A., Hill P.S. et al. Community health extension program of Ethiopia, 2003–2018: successes and challenges toward universal coverage for primary healthcare services. Global Health 15, 24 (2019). doi: 10.1186/s12992-019-0470-1 30914055 PMC6434624

[40] Gudina EK, Gobena D, Debela T, et al COVID-19 in Oromia Region of Ethiopia: a review of the first 6 months’ surveillance data BMJ Open 2021;11:e046764. doi: 10.1136/bmjopen-2020-046764 33782023 PMC8008954

[41] Baye KA. COVID-19 prevention measures in Ethiopia Current realities and prospects MAY,2016.

